# Managing Comorbidities, Determinants and Disability at Start and End of TB Treatment under Routine Program Conditions in China

**DOI:** 10.3390/tropicalmed8070341

**Published:** 2023-06-26

**Authors:** Yuhong Liu, Yan Lin, Yuxian Sun, Pruthu Thekkur, Changhao Cheng, Yuecui Li, Yunzhen Shi, Jun Jiang, Jiong Liao, Chuangui Nie, Wenyan Sun, Chengyuan Liang, Xiaojuan Zhang, Sang Liu, Yan Ma, Selma Dar Berger, Srinath Satyanarayana, Ajay M. V. Kumar, Mohammed Khogali, Rony Zachariah, Jonathan E. Golub, Liang Li, Anthony D. Harries

**Affiliations:** 1 Beijing Chest Hospital, Capital Medical University, No. 9 Beiguan Ave, Tongzhou, Beijing 101149, China; liuyuhong0516@126.com (Y.L.); yuxian960224@yeah.net (Y.S.); liliang@tb123.org (L.L.); 2 Beijing Tuberculosis and Thoracic Tumor Research Institute, Beijing 101149, China; 3 International Union Against Tuberculosis and Lung Disease, 2 Rue Jean Lantier, 75001 Paris, France; ylin.consultant@theunion.org (Y.L.); pruthu.tk@theunion.org (P.T.); sberger@theunion.org (S.D.B.); ssrinath@theunion.org (S.S.); akumar@theunion.org (A.M.V.K.); 4 The Union South-East Asia Office, C-6 Qutub Institutional Area, New Delhi 110016, India; 5 Wuhan Pulmonary Hospital, No. 28 Baofengyilu, Qiaokou, Wuhan 430000, China; chengchanghao@126.com; 6 The First People’s Hospital of Yongkang, No. 599 Jinshan West Road, Yongkang 321300, China; yklycwh@126.com; 7 Dongyang People’s Hospital, No. 60 Wuning West Road, Dongyang 322100, China; syzwylwmc@163.com; 8 The Third People’s Hospital of Yichang City, No. 32 Gangyaolu, Yichang 443000, China; jj731218@163.com; 9 The People’s Hospital of Laiban, No. 159 Pangudadao, Laiban 546100, China; lbsrmyygrk2023@163.com; 10 Xiangyang Institute of Tuberculosis Control and Prevention, No. 20 Xinhuala, Xiangyang 441000, China; huanbshi@163.com; 11 Ezhou Third Hospital, No. 16 Minxin West Road, Ezhou 436000, China; 17707116436@163.com; 12 Baise City People’s Hospital, No. 8 Chengxianglu, Youjiang, Baise 533000, China; yian_584@msn.com; 13 Zhongwei People’s Hospital, Gulouxijie, Zhongwei 755000, China; quqibinggan123@163.com; 14 Guangxi Chest Hospital, No. 8 Yangjiaoshanlu, Yufeng, Liuzhou 545000, China; is7978@163.com; 15 The People’s Hospital of Tongxin, Xueyuanlu, Tongxi, Yuhaizhen 751100, China; 13195077234@163.com; 16 Yenepoya Medical College, Yenepoya (Deemed to be University), Mangalore 575018, India; 17 Institute of Public Health, College of Medicine and Health Sciences, United Arab Emirates University, Al Ain 17666, United Arab Emirates; ahmedm@uaeu.ac.ae; 18 UNICEF, UNDP, World Bank, World Health Organization, Special Programme for Research and Training in Tropical Diseases (TDR), Avenue Appia 20, 1211 Geneva, Switzerland; zachariahr@who.int; 19 Johns Hopkins Center for Tuberculosis, Johns Hopkins University School of Medicine, Baltimore, MD 21205, USA; jgolub@jhmi.edu; 20 Department of Clinical Research, Faculty of Infectious and Tropical Diseases, London School of Hygiene and Tropical Medicine, Keppel Street, London WC1E 7HT, UK

**Keywords:** tuberculosis, China, diabetes mellitus, high blood pressure, mental health disorder, cigarette smoking, excess alcohol drinking, SORT IT, 6 min walking test, disability

## Abstract

Many patients with tuberculosis (TB) have comorbidities, risk determinants and disability that co-exist at diagnosis, during and after TB treatment. We conducted an observational cohort study in 11 health facilities in China to assess under routine program conditions (i) the burden of these problems at the start and end of TB treatment and (ii) whether referral mechanisms for further care were functional. There were 603 patients registered with drug-susceptible TB who started TB treatment: 84% were symptomatic, 14% had diabetes, 14% had high blood pressure, 19% smoked cigarettes, 10% drank excess alcohol and in 45% the 6 min walking test (6MWT) was abnormal. Five patients were identified with mental health disorders. There were 586 (97%) patients who successfully completed TB treatment six months later. Of these, 18% were still symptomatic, 12% had diabetes (the remainder with diabetes failed to complete treatment), 5% had high blood pressure, 5% smoked cigarettes, 1% drank excess alcohol and 25% had an abnormal 6MWT. Referral mechanisms for the care of comorbidities and determinants worked well except for mental health and pulmonary rehabilitation for disability. There is need for more programmatic-related studies in other countries to build the evidence base for care of TB-related conditions and disability.

## 1. Introduction

Worldwide, tuberculosis (TB) remains a major public health problem and is one of the top 10 causes of death overall [1]. Despite a gradual fall in TB incidence and TB mortality over the last 20 years, in 2021 there were 10.6 million new cases of the disease and 1.6 million deaths [1].

Treatment success rates of 90% or higher have been proposed for patients with all types of TB to help achieve the World Health Organization (WHO) and Sustainable Development Goal (SDG) targets of ending TB by 2030. In 2020, there was an overall global TB treatment success of 86% for the cohort of patients enrolled on first-line TB treatments [1]. While commendable, this achievement takes no account of post-TB pulmonary or extrapulmonary complications, which negatively affect quality of life of TB survivors. Indeed, for many decades international guidelines have failed to address the issue of the post-TB health of TB survivors despite a growing body of evidence demonstrating increased morbidity, mortality and disability of patients after completion of TB treatment [2,3,4]. As such, post-TB health has historically not been a focus in the TB control agendas of most national TB Programs [3].

It is well established that a significant proportion of successfully treated TB patients continue to suffer from TB-related functional disability [5,6,7]. A systematic review and meta-analysis of nearly 220,000 TB survivors estimated the prevalence of post-TB mental health disorder at 23%, respiratory dysfunction at 21% and musculoskeletal impairment at 17% [8]. It was recently estimated, using disability-adjusted life years (DALYs) to summarize fatal and non-fatal health losses attributable to TB, that 47% of the 122 million DALYs due to global incident TB in 2019 were due to post-TB sequelae [9].

As an important component of a patient-centered service, it is recommended that TB patients should be screened and assessed for comorbidities, risk determinants and disabilities at the start and end of TB treatment, and those identified with problems should receive appropriate care and support [10]. Furthermore, those patients with impaired lung function should be referred for pulmonary rehabilitation as early as possible in order to prevent post-TB sequelae [11,12].

Most national TB programs (NTP) focus only on TB diagnosis and administration of treatment that is restricted to the duration when patients are on such treatment. There is a dearth of evidence about how post-TB assessments could be carried out in routine healthcare settings. In this regard, the first study in China was conducted in 2021 and assessed the feasibility and value of conducting post-TB assessments in patients consecutively completing TB treatment within a routine programmatic setting [13]. The assessment was successfully conducted within the routine programmatic setting and health care workers found it feasible and useful to undertake their work. Implementation, however, was small scale, involving only five clinics and hospitals, and did not include assessments at the start of TB treatment.

We therefore conducted an operational research study, using the Structured Operational Research and Training Initiative (SORT IT) model [14], in 11 clinics and hospitals in China and included assessments for comorbidities, risk determinants and functional disability in patients registered for TB treatment at the start and at the end of TB treatment. There were two specific objectives. The first objective was to assess patients consecutively registered over a period of 3 to 4 months with drug-susceptible TB at two time points: at the start and end of TB treatment. The assessment comprised a structured questionnaire and targeted investigations that documented symptoms, signs, important risk determinants for TB, selected comorbidities and disability using the 6 min walking test (6MWT). The second objective was to refer patients with identified abnormalities, determinants and comorbidities to appropriate care and those with poor 6MWT for a pulmonary rehabilitation package. Two months after referral from TB registration and one month after the end of TB treatment, patients were assessed as to whether they had been referred to care services and started care.

## 2. Materials and Methods

### 2.1. Study Design

This was a cohort study carried out within the routine health services in China.

### 2.2. Sites and Setting

**Figure 1:**
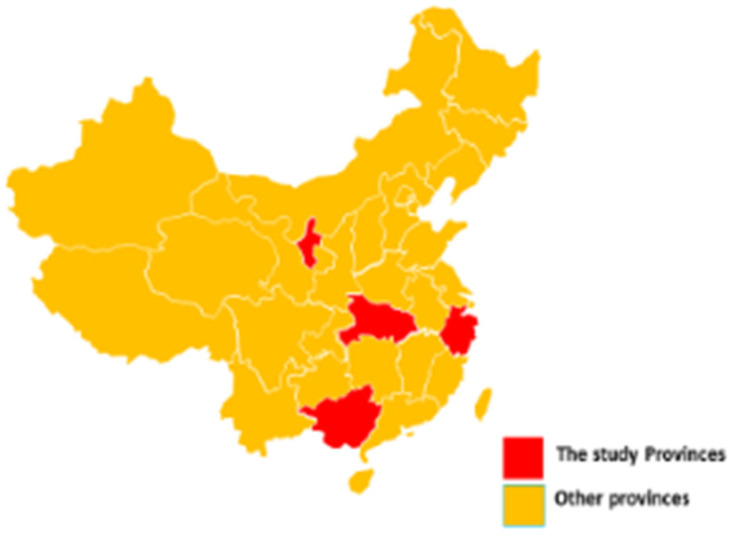
Location of the provinces in China where the study was implemented.

Previous experience of assessing TB patients at the end of TB treatment in 2021 [13] led to a decision that the study should be carried out in patients from urban and rural settings. Therefore, health facilities that looked after TB patients across mainland China were selected. Locations of the provinces of the 11 health facilities are shown in Figure 1 and details of the 11 health facilities, including whether their catchment areas were from urban and/or rural settings, are shown in Table 1. The selection was purposive and based on geographical coverage, broadly defined economic development levels of the catchment areas, sufficient numbers of people with TB registered each year and willingness of staff to participate in the study without requirements of additional funding support. All 11 health facilities had on-site chest radiographic facilities, a laboratory for blood tests such as fasting blood glucose and equipment such as blood pressure machines.

### 2.3. Study Population

The study population included people aged 15 years and above who were diagnosed with TB and consequently registered in one of the 11 hospitals or clinics selected for our study between March and July 2022. All TB patients in China are diagnosed based on clinical examination, sputum examination for smear microscopy and molecular tests (Xpert MTB/RIF assay) and a chest radiograph. In our study, all patients had drug-susceptible TB or no confirmed evidence of drug resistance, and they were registered to receive the 6-month standard TB treatment regimen consisting of rifampicin, isoniazid, pyrazinamide and ethambutol for the initial two months, followed by rifampicin and isoniazid for the four months of the continuation phase. Patients who were terminally ill and not expected to survive beyond 6 months were not included. Each health facility was advised to recruit up to 60 patients within the time period and to stop further recruitment if this number was reached.

### 2.4. Training Health Professionals Who Were Involved in this Study

Following the launch meeting of the project, a two-hour online training was jointly organized by the International Union Against Tuberculosis and Lung Disease (The Union) and the Tuberculosis Clinical Center of China Centers for Disease Control and Prevention (CDC). The health professionals who were involved in this study received training on the reasons for patient assessments and how to conduct the assessments, including how to complete the evaluation form and how to conduct the 6MWT. They also received training on the principles and practice of operational research and how to write a scientific paper as part of the capacity building component of this project.

### 2.5. Assessment for Comorbidities, Risk Determinants and Disability

A simplified evaluation form was developed based on the nature of the disease and the potential lung damage caused by TB, the potential lifelong comorbidities and measures outlined in the WHO Policy on Prevention of Cardiovascular Disease [15]. The evaluation form was translated into Chinese and sent to the hospital and clinics for the on-site evaluations. The actual assessment consisted of four steps or components:

#### 2.5.1. At the Time of TB Registration

Patients were assessed for selected comorbidities, risk determinants and disability before starting TB treatment. The comorbidities included diabetes mellitus (DM), high blood pressure and mental health disorders. Patients were asked whether they had a known diagnosis of DM. For all patients, with known DM or not, a fasting blood glucose (FBG) was performed and an FBG ≥ 7.0 mmol/L (≥126 mg/dL) was defined as abnormal. New patients with FBG ≥ 7.0 mmol/L were referred to a diabetes clinic for confirmation of DM. Patients were screened for high blood pressure and any person with systolic blood pressure ≥ 140 mmHg and/or diastolic blood pressure ≥ 90 mmHg was diagnosed as having high blood pressure. Mental health was initially assessed by TB staff by asking if there was a known diagnosis of mental health disorder and/or if the patient was suffering from any mental health issues. Any patient suspected of having a mental health disorder was referred to a specialist in the mental health clinic for confirmation.

The risk determinants included cigarette smoking, excess alcohol intake, exposure to silica dust, use of recreational drugs and malnutrition. Cigarette smoking was defined as the daily use of any kind of tobacco product currently or within the last six months. Excess alcohol intake was defined as daily alcohol consumption comprising spirits and/or a large bottle of beer (600–750 mL). Silica exposure was defined as those currently exposed professionally to silica dust (for example, miners). Persons were asked whether they used recreational drugs. A diagnosis of malnutrition for this study was based on body mass index (BMI-weight in kg/height ^2^ in meters) < 18.0. We took a BMI threshold slightly below the WHO standard of 18.5 so as not to overwhelm referral to nutrition support services at the start of TB treatment, given that TB is an important cause of malnutrition, and to make it an easier cut-off to use for frontline staff.

Those with a comorbidity/risk determinant were referred to the appropriate service for care and attention either at the same health facility where the diagnosis of TB was made or at a different health facility, depending on services offered at the facilities.

Disability was assessed by a 6MWT performed under supervision and according to the local environment. For those hospitals or clinics with a large garden or open square, the attending physician or nurse directed the patients to walk around a track for six minutes using a mobile phone or simple watch to measure the time. For a clinic without this facility, the 6MWT was performed on the side of the street and patients were directed to walk back and forth on a measured track. Distance walked in meters was measured using a pedometer or a known length of track. Based on the previous study in China [13], and based on a study in seven countries in healthy adults [16], an abnormal 6MWT was defined as walking < 400 m in six minutes. For the initial assessment, the time taken for the health worker to perform the questionnaire assessment and 6MWT was measured. Each health worker was also asked about whether there were any challenges with conducting these initial assessments under routine program conditions.

#### 2.5.2. At the End of the Initial 2 Months of TB Treatment

The attending physician or nurse followed up with patients when they attended the health facilities for monitoring of their TB treatment to see if those eligible for referral had been referred and had received the appropriate care, and they updated this information on the evaluation checklist. The care included: receiving specific advice, confirmation of diagnosis and/or treatment offered by specialists in a specialized institution or in the same institution. This assessment was done through self-report and verified through care records.

#### 2.5.3. At the End of TB Treatment

Patients who had successfully completed TB treatment were reassessed in the same manner as for Step Section 2.5.1. and those with identified problems were referred to appropriate care.

#### 2.5.4. At One Month after the End of TB Treatment

The same attending physician or nurse followed up those patients with comorbidities, risk determinants or disability to determine whether they had received appropriate care through self-report and case record verification as explained in Section 2.5.2.

### 2.6. Data Collection, Analysis and Statistics

A pre-designed data entry software (iTrial, Imedding Technology Company Limited, Beijing, China) was used for data collection. Individual patient data were entered into the software by the attending physician or nurse and cross-checked by the Tuberculosis Clinical Centre staff and the Union consultant in Beijing. Categorical data were presented as frequencies and proportions and continuous data as means and standard deviations (SD). Comparisons between groups were made using the chi-square test (or Fisher’s exact test with small numbers) with levels of statistical significance set at 5% (*p* < 0.05).

## 3. Results

### 3.1. Characteristics of Registered TB Patients

There were 603 patients with TB registered for TB treatment in the 11 hospitals and clinics between March and July 2022. Their characteristics are shown in Table 2.

The age of the patients ranged from 15 to 93 years with a mean of 54.4 ± 17.7 years. Of these, the majority were male, living in rural areas, and with pulmonary TB, which was bacteriologically confirmed in more than half of cases. Of the 10 patients with extra pulmonary TB, lymphadenopathy was diagnosed in five, pericarditis in one, peritonitis with pericarditis in one, genitourinary TB in one, gastrointestinal TB in one and spinal TB in one. Over 90% of the patients had been previously treated for TB. Almost all patients had radiographic abnormalities, with bilateral disease in about two thirds and cavities in nearly one quarter.

### 3.2. Comorbidities, Risk Determinants and Disability

The symptoms, comorbidities, risk determinants and disability of TB patients at the time of registration and at the end of TB treatment are shown in Table 3.

#### 3.2.1. At TB Registration

While 84% of patients were symptomatic, the remainder had no symptoms and were diagnosed on the basis of physical examination and chest radiography. The majority of those with symptoms had a cough and/or shortness of breath.

There were 85 (14%) patients with DM; this included 79 with an already known diagnosis (regardless of their FBG level) and 6 with newly diagnosed DM at this visit. FBG ≥ 7.0mmol/L was found in 74 patients. Of these, 59 had known DM, 6 were newly diagnosed with DM (after confirmation of the FBG at the DM clinic) and 9 had no DM diagnosis made after being referred for confirmatory testing to the DM clinic. There were 82 (14%) patients identified with high blood pressure, with high systolic blood pressure being more common than diastolic blood pressure. Only five patients were identified with a confirmed mental health disorder.

There were 113 (19%) cigarette smokers, 60 (10%) who drank excess alcohol and 137 (23%) whose BMI was <18. A few patients had current exposure to silica dust as a result of mining occupations and no one reported use of recreational drugs.

The 6MWT was carried out in 576 patients. The test was not done in the remaining 27 patients because they were in poor general health, were amputees or had other foot problems that prevented them from walking. Of 576 patients performing the 6MWT, the mean (SD) distance walked was 402.7 ± 128.0 m. Of these patients, 258 (45%) walked <400 m in 6 min. For those who walked <400 m, there were no significant associations between patients with or without diabetes (*p* = 0.38), between patients living in rural areas compared with urban areas (*p* = 0.92) or between patients with and without radiographic cavities (*p* = 0.43). However, significantly more patients who were non-smokers walked <400 m (224/490, 45.7%) compared with patients who were smokers (34/113, 30.0%)-*p* < 0.01).

The mean (SD) time taken to complete the assessment questionnaire and 6MWT was 17.2 ± 21.8 min. No staff member reported any major challenges with conducting the assessments.

#### 3.2.2. At End of TB Treatment

There were 586 (97%) patients who successfully completed TB treatment (they were either cured with negative sputum smears at the end of treatment or they completed treatment without sputum smear evaluation). Of these, 105 (18%) remained symptomatic, with cough being the most common symptom.

There were 73 (12%) patients with DM who successfully completed TB treatment; these 73 had an already known diagnosis, which includes those newly diagnosed at registration (12 of the original 85 patients with DM at registration failed to successfully complete treatment). There were no newly diagnosed DM patients identified at the end of TB treatment. An FBG ≥ 7.0mmol/L was found in 50 patients; of these, 40 had known DM, there were no newly diagnosed patients with DM and in 10 no DM diagnosis was made after being referred for confirmatory tests to the DM clinic. There were 32 (6%) patients identified with high blood pressure, with high systolic blood pressure being as common as high diastolic blood pressure. There were four patients identified with a confirmed mental health disorder, one of these being newly diagnosed at the end of treatment with schizophrenia.

There were 27 (5%) cigarette smokers (meaning that 86 quit during treatment), 6 (1.0%) who drank excess alcohol and 95 (16%) whose BMI was <18. There were five patients who had current exposure to silica dust as a result of mining and no one reported use of recreational drugs.

The 6MWT was carried out on 557 patients. The test was not done on the remaining 29 patients for the same reasons as at the time of registration. Of those who performed the 6MWT, the mean (SD) distance walked was 463.8 ± 125.8 m and 137 (25%) walked <400 m. With respect to walking <400 m, there were no significant associations with gender (*p* = 0.12) or patients being with or without diabetes (*p* = 0.24). However, significantly more patients who were non-smokers walked <400 m (136/559, 24.3%) compared with patients who were smokers (1/27, 3.7%)—*p* = 0.012, and significantly more patients aged ≥60 years walked <400 m (99/233, 42.5%) compared with those aged < 60 years (38/324, 11.7%)—*p* < 0.001.

The time taken to perform the assessment and 6MWT was not measured at the end of anti-TB treatment.

#### 3.2.3. Patients with Associated Problems at Both the Start and the End of TB Treatment

Patients with symptoms, comorbidities, risk determinants and disability at the start and end of TB treatment are shown in Table 4. The majority of those with symptoms, especially cough, at the end of TB treatment had the same symptoms at the start of treatment. All of those who were smokers, drank excess alcohol or had exposure to silica at the end of treatment had these same problems at the start of treatment. However, with high blood pressure, mental health disorder and malnutrition, between 25–45% of new patients developed these problems at the end of treatment and did not have them at the start. With respect to an abnormal 6MWT, 90% of those walking <400 m at the end of TB treatment could also not walk 400 m or more at the start of treatment.

### 3.3. Referrals for Care in TB Patients with Comorbidities, Risk Determinants or Disability

#### 3.3.1. Referral and Receipt of Care at the Time of Registration

The referral systems for care in TB patients identified with comorbidities, risk determinants and disability at the time of registration and two months later are shown in Table 5. Small numbers of patients with DM, high blood pressure and exposure to silica were already receiving care at the time of registration.

Of those needing referral to care, almost 100% of those with comorbidities and risk determinants were referred, with the majority being referred within the same facility in which they were receiving TB treatment. The exception was for those with an abnormal 6MWT, of whom <5% were referred.

Between 75–100% of those referred to care were in receipt of care when assessed two months later. However, with mental health disorders, only one of five persons was in receipt of care at this time.

#### 3.3.2. Referral and Receipt of Care at the End of TB Treatment

The referral systems for care in TB patients identified with comorbidities, risk determinants and disability at the end of TB treatment and one month later are shown in Table 6. Small numbers with DM, high blood pressure and malnutrition were already receiving care at the end of TB treatment.

Of those needing referral to care, 100% of those with comorbidities, risk determinants and disability were referred, with most of those again receiving care within the same facility.

For those with DM and high blood pressure, about 90% of those referred were in receipt of care one month later. With mental health disorders, no person received care. For those with risk determinants, between 85–100% of patients were in receipt of care. Of those with disability, only 23 (17%) patients were in receipt of care one month later.

## 4. Discussion

This is the first time in China that patients diagnosed with TB have been assessed in the routine program setting for symptoms, comorbidities, risk determinants and disability at the start and end of TB treatment as well as tracked for referral to care. There were seven key findings.

First, similar to the study on post-TB assessment conducted two years ago [13], we found it was feasible to conduct these assessments at the start and end of TB treatment without additional resource needs or incentives. The average time taken to complete the first round of questionnaires plus the 6MWT was a little shorter at 17 min compared with the first study [13].

Second, the cohort of TB patients who were consecutively registered were similar in their socio-demographic and clinical profile to cohorts in other parts of the world [1], with the exception that the majority had previous TB. Many of these patients had their recurrent episode of TB clinically diagnosed based on symptoms, signs and radiographic findings, and it is possible that some of them had a disease other than TB. Nonetheless, they were all registered and treated for TB within the national program setting. The finding of previous TB in the majority of our patients was surprising, there is no clear explanation, and it requires further investigation. While the majority of patients were symptomatic at registration, there was a substantial proportion who were asymptomatic. Diagnosis in these patients was on the basis of physical checks and chest radiography for entry to school, university or specific occupations. Asymptomatic patients were particularly frequent in Laibin City Hospital where an active case finding campaign was in progress at the time. By the end of treatment, 18% of patients remained symptomatic which was far fewer than the 47% with on-going symptoms found in the previous study in China [13]. This applied to all the symptoms (cough, shortness of breath and fatigue). Reasons for these differences are unclear but fewer patients were smoking at the end of treatment compared with the previous study and this may have contributed to the reduction in symptoms.

Third, at the time of registration, comorbid conditions of DM and high blood pressure were found in 10–15% of the patients. As a result of urbanization, improved life expectancy and better access to medical services, chronic comorbid non-communicable diseases are becoming more common in patients with TB [3,17] and this is particularly the case in China [18]. In this respect, an important associated comorbidity is DM. The prevalence of DM in TB patients in our study was similar to what has been found previously in China [19], emphasizing the importance of screening for this disease which not only increases the risk of TB but negatively impacts treatment outcomes. People with both TB and DM are less likely to recover from TB and more likely to die, they suffer an increased risk of recurrent TB after treatment completion, have a poorer quality of life and they are also more likely to develop multidrug-resistant TB [20,21]. The fact that 12 of the 85 patients with DM and TB at registration failed to successfully complete treatment exemplifies this point. Unlike DM, high blood pressure is not associated with an increased risk of developing TB but there is evidence to suggest that it increases mortality during and after TB treatment [22] and it therefore warrants screening.

Fourth, we found few patients with mental health disorders in contrast to a systematic review that identified mental health disorders in nearly a quarter of TB patients [8]. Anxiety and depression seem to be particularly common if patients are properly screened [23,24]. In our study we did not use a systematic screening tool such as the validated Patient Health Questionnaire (PHQ-9) [25] and it is likely that we underestimated the prevalence of mental health disorder.

Fifth, at the time of TB registration, and according to our definitions, nearly 20% and 10% of TB patients smoked cigarettes and drank excess alcohol, respectively. Both cigarette smoking and excess alcohol drinking are well known risk factors for TB [26,27]. Smoking is also associated with poor treatment outcomes and an increased risk of TB recurrence after completion of treatment [28,29], and alcohol use similarly increases the risk of death, treatment failure and loss to follow-up during TB treatment [30]. In our study, almost all patients who smoked and drank excess alcohol were referred to care soon after registration, with the majority still in care two months later. At the end of TB treatment only a small number continued to smoke or drink excess alcohol. Previous reports have suggested that it is feasible and effective to integrate smoking cessation into TB programs [31], and the findings of our study endorse this. The same principles apply to excessive alcohol consumption [32].

Sixth, malnutrition is an established risk factor for TB with a dose–response relationship observed between decreasing BMI and increasing risk of TB [33,34]. TB, however, also causes weight loss, which can be addressed and corrected solely with TB treatment [35]. One quarter of our patients had a low BMI at registration with the prevalence of low BMI substantially reduced at the end of treatment. All patients with malnutrition at the start of TB treatment were referred to care, but whether it was the referral or the effect of TB treatment or both that made the difference is unclear. Nutritional counselling and food support, however, is a desirable intervention during TB treatment, and in one recent study in Africa it was associated with reduced loss to follow-up [36].

Seventh, disability (assessed by a 6MWT < 400 m) was observed in nearly half of the TB patients at the start of TB treatment and about one quarter of patients at the end. While it was understandable that older patients at the end of TB treatment had a higher frequency of functional disability compared with younger patients, we found it surprising that non-smokers had a higher frequency of disability than smokers. We wonder if this latter association may have been spurious and due to the small number of participants. As with the previous study in China [13], the lack of traditional lung function equipment and pulmonary spirometry led us to assess disability using the 6MWT. A study in India attested to its simplicity and value in predicting mortality in patients with chronic lung disease, including TB [37], and it has been used elsewhere to assess lung function status in post-TB patients [38].

At the start of anti-TB treatment, few patients with a 6MWT < 400 m were referred for pulmonary rehabilitation, the main reason being the reluctance of rehabilitation staff to be exposed to infectious TB patients. In contrast, at the end of TB treatment, all the patients with an abnormal 6MWT were referred and accepted for rehabilitation although less than 20% were in receipt of care one month later. We did not assess what type of rehabilitation services were provided. Simple pulmonary rehabilitation programs in Europe, Africa and Asia, supervised by physiotherapists and other staff, can make a notable difference and can lead to improvement of quality of life, exercise tolerance and respiratory outcomes [39,40].

This study had some notable strengths that included its large sample size, implementation within the routine programmatic setting and the conduct and reporting of the study in line with Strengthening the Reporting of Observational Studies in Epidemiology (STROBE) guidelines [41].

There were, however, some limitations. First, we excluded patients who were judged by clinical staff to be terminally ill. The number of excluded patients was not documented. However, this explains the high treatment success in those enrolled in the study and probably means we underestimated the magnitude of comorbidities, risk determinants and disability. Our sample size also was not adequate to examine associations in an adjusted analysis.

Second, our assessments of some comorbidities and risk determinants could have been better. High blood pressure was documented based on a one-time measurement and we did not ask patients whether they were already on treatment for known hypertension. We did not use the PHQ-9 score to assess mental health disorder as we felt it would take too long, but we could have used the shorter but equally valuable PHQ-2 score [42]. Similarly, our assessment of excess alcohol drinking based on daily spirit or large volume beer intake is not evidence based, but we again felt that the ten-item questionnaire of the WHO-based alcohol use disorder identification test (AUDIT) [43] would take too long and be too cumbersome for routine use in these busy TB clinics.

Third, the 6MWT was performed according to the local situation in the study sites and we did not use a standardized facility as suggested by the American Thoracic Association [44]. This may have caused some measurement differences in the 6MWT between health facilities. We also did not systematically capture the reasons why a small number of patients did not perform the 6MWT, although we know that the majority were unable to walk.

Fourth, we did not fully investigate the type or quality of care offered to patients who were referred to the appropriate health services, and this applied particularly to those referred for pulmonary rehabilitation.

Despite these limitations, our findings have important policy and practical implications. First, we have demonstrated that it is feasible and useful in China to assess comorbidities, risk determinants and disability at the start and the end of TB treatment and to refer those with problems to appropriate care. This fully aligns with the Lancet Commission on building a TB-free world where the focus must be on patient-centered services to improve the quality and delivery of care for TB, for TB-related disability and for its associated comorbidities [45]. We now need to demonstrate feasibility and usefulness in other countries.

Second, this study was focused on adults. There is growing evidence that TB in children has an adverse impact on subsequent physical growth, and in the years after TB has been successfully treated the disease is also associated with an increased frequency of childhood wheezing and with both obstructive and restrictive lung disease [46]. More programmatic work needs to be done to evaluate and support children at the start and end of TB treatment.

Third, moving forward we need to develop simple tools to identify comorbidities, determinants and disability that can allow an easy and quick assessment within the busy program setting and yet provide a reliable and consistent measure of each problem. The 6MWT in particular needs to be better standardized for use in low- and middle-income countries.

Fourth, we need to work out how and where best to offer care for the problems we identify. In particular, access globally to pulmonary rehabilitation is poor, with less than 3% of patients with chronic lung disease being able to access this type of service [47].

Finally, we need to follow up these patients longer to determine whether quality of life does improve, and from a TB viewpoint, whether this helps to reduce the frequency of recurrent TB and thus the overall burden of TB.

## 5. Conclusions

This study, carried out under routine program settings in 11 health facilities in mainland China, showed that it was feasible and useful to assess TB patients for symptoms, selected comorbidities, risk determinants and disability at the start and at the end of TB treatment. The prevalence of these conditions was less frequent at the end compared with the beginning of treatment. Referral of patients with various conditions to care at both the start and end of TB treatment worked well, except for those with mental health disorders and disability, where fewer patients were found to be in receipt of care compared with the others. Moving forward, more programmatic-related studies need to be conducted in other countries to build the evidence base, more needs to be done to simplify the assessment tools and resources need to be mobilized to strengthen referral mechanisms and provision of care.

## Figures and Tables

**Table 1:** Characteristics of the 11 clinics/hospitals in China selected for TB patient assessments.

Name of Hospital or Clinic	Location City/County	General Population Served	Number of TB Patients Registered in 2020	Main Catchment (Urban/Rural)
Wuhan Pulmonary Hospital	Wuhan, Hubei	57,750,000	4938	Urban
Xiangyang Institute of Tuberculosis Control & Prevention	Xiangyang, Hubei	1,420,000	607	Urban
Ezhou Third Hospital	Ezhou, Hubei	1,000,000	560	Urban
Guangxi Chest Hospital	Liuzhou, Guangxi	4,160,000	3559	Urban/rural
The Third People’s Hospital of Yichang City	Yichang, Hubei	4,000,000	808	Urban/rural
The People’s Hospital of Laibin	Laibin, Guangxi	1,000,000	636	Urban/rural
Baise City People’s Hospital	Baise, Guangxi	470,000	345	Urban/rural
Zhongwei People’s Hospital	Zhongwei, Ningxia	400,000	127	Urban/rural
The People’s Hospital of Tongxin	Tongxin, Ningxia	320,000	121	Rural
Dongyang People’s Hospital	Dongyang, Zhejiang	1,080,000	428	Rural
First People’s Hospital of Yongkang City	Yongkang, Zhejiang	960,000	477	Rural

**Table 2:** Demographic and clinical characteristics of registered TB patients in the 11 hospitals/clinics in China between March and July 2022.

Category	Variable	Number	(%)
Registered TB patients	Total	603	
Gender	Male	401	(66.5)
	Female	202	(33.5)
Hospital / Clinic	Zhongwei	49	(8.1)
	Tongxin	34	(5.6)
	Baise	53	(8.8)
	Guangxi	46	(7.6)
	Laiban	60	(10.0)
	Dongyang	60	(10.0)
	Yongkang	60	(10.0)
	Ezhou	60	(10.0)
	Wuhan	60	(10.0)
	Xiangyang	60	(10.0)
	Yichang	61	(10.0)
Community location	Urban	232	(38.4)
	Rural	371	(61.6)
Type of Disease	Pulmonary TB	593	(98.4)
	Extrapulmonary TB	10	(1.6)
Bacteriology status	Bacteriologically confirmed	350	(58.0)
	Clinically diagnosed	253	(42.0)
Category of TB	New	55	(9.2)
	Retreatment	548	(90.8)
Chest radiography ^A^	Chest radiograph done	603	(100)
	Chest radiograph abnormal	599	(99.3)
	Bilateral abnormalities	409	(67.8)
	Unilateral abnormalities	154	(25.5)
	Cavities	135	(22.4)
	Radiographic shadows	54	(9.0)
	Shrinkage	15	(2.5)
	Scarring	1	(0.2)
	Other	41	(6.8)

Footnotes: percentages are those of total numbers; TB = tuberculosis. ^A^ = some patients had multiple chest radiographic abnormalities.

**Table 3:** Symptoms, comorbidities, determinants and functional disability at TB registration and end of TB treatment for those successfully completing TB treatment in 11 clinics/hospitals in China.

Category	Variable	TB Registration		End of TB Treatment	
		Number	(%)	Number	(%)
Total cohort		603		586	
	No symptoms	98	(16.3)	481	(82.0)
	Symptomatic	505	(83.7)	105	(17.9)
	Cough	396	(65.7)	43	(7.3)
	Shortness of Breath	133	(22.1)	20	(3.4)
	Fatigue	105	(17.4)	39	(6.7)
	Chest pain	80	(13.3)	4	(0.7)
	Other	82	(13.6)	29	(4.9)
Co-morbidities	Diabetes Mellitus: ^A^	85	(14.1)	73	(12.4)
	Known Diabetes (DM) diagnosed elsewhere	79	(13.1)	73	(12.4)
	New Diabetes (DM) diagnosed on this visit	6	(1.0)	0	(0)
	High blood pressure: ^B^	82	(13.6)	32	(5.5)
	Systolic Blood Pressure ≥140 mmHg	65	(10.8)	20	(3.4)
	Diastolic Blood Pressure ≥90 mmHg	45	(7.5)	21	(3.6)
	Mental Health Disorder:	5	(0.8)	4	(0.7)
	Depression	2	(0.3)	1	(0.2)
	Anxiety	1	(0.2)	1	(0.2)
	Schizophrenia	1	(0.2)	2	(0.3)
	Hysteria	1	(0.2)	0	(0)
Risk determinants	Cigarette smoker	113	(18.7)	27	(4.6)
	Excess alcohol drinking	60	(10.0)	6	(1.0)
	Current exposure to silica dust	14	(2.3)	5	(0.9)
	Use of recreational drugs	0	(0)	0	(0)
	Malnutrition (BMI < 18)	137	(22.6)	95	(16.2)
Disability	6MWT done	576	(95.5)	557	(95.1)
	6MWT < 400 m ^C^	258	(44.8)	137	(24.6)

Footnotes: Percentages are those of total numbers. TB = tuberculosis; BMI = body mass index (weight kg/height m^2^); 6MWT = 6 min walking test. ^A^ = patients with known DM and patients newly diagnosed with DM. ^B^ = some patients had high systolic and diastolic blood pressure. ^C^ = denominator is number having 6MWT done.

**Table 4:** The number of TB patients with symptoms, comorbidities, risk determinants and disability at the end of TB treatment who also had these problems at the time of TB registration in 11 clinics/hospitals in China.

Category	Variable	Patients with the Problem at the End of TB Treatment	The Same Patients with the Problem at the Start of TB Treatment	
		Number	Number	(%)
Symptoms	Symptomatic	105	103	(98.1)
	Cough	43	40	(93.0)
	Shortness of breath	20	15	(75.0)
Comorbidities	New Diabetes	0 ^A^	0	
	High blood pressure	32	23	(71.9)
	Mental Health Disorder	4	3	(75.0)
Determinants	Cigarette smoker	27	27	(100)
	Excess alcohol drinking	6	6	(100)
	Exposure to silica	5	5	(100)
	Malnutrition	95	52	(54.8)
Disability	6MWT < 400 m	137	123	(89.8)

Footnotes: ^A^ at the end of treatment, all the newly diagnosed persons with diabetes had been reclassified as “known diabetes”. Of the six persons newly diagnosed with diabetes at registration, five completed TB treatment and one did not.

**Table 5:** Performance of the referral systems for care in TB patients identified with co-morbidities, risk determinants and disability at the time of TB registration.

Category	Variable	At Registration	Already Receiving Regular Care	Need for Referral to Care	Referred to Care		Referred to Care		In Receipt of Care Two Months after Registration	
							Elsewhere in Another Facility	On Site at Same Facility		
		Number	Number	Number	Number	(%) ^A^	Number	Number	Number	(%) ^B^
Total cohort	TB Registration	603	162	456	294	(64.5)	32	262	255	(86.7)
Co- morbidities	Diabetes Mellitus	85	10	75	75	(100)	9	66	61	(81.3)
	Previous Diabetes	79	10	69	69	(100)	6	63	61	(88.4)
	New Diabetes	6	0	6	6	(100)	3	3	6	(100)
	High blood pressure	75	14	61	61	(100)	11	50	47	(77.0)
	MHD	5	0	5	5	(100)	3	2	1	(20.0)
Risk Determinants	Cigarette smoker	113	0	113	113	(100)	0	113	102	(90.3)
	Excess alcohol use	60	0	60	59	(98.3)	0	59	50	(84.8)
	Exposure to silica	14	5	9	9	(100)	9	0	9	(100)
	Malnutrition	137	0	137	137	(100)	0	137	137	(100)
Disability	6MWT < 400 ^C^	258	0	258	12	(4.7)	2	10	9	(75.0)

Footnotes: TB = tuberculosis; MHD = mental health disorder; Malnutrition defined as body mass index < 18.0; 6MWT = 6 min walking test. ^A^ = denominator is number of patients who needed referral to care. ^B^ = denominator is number of patients who were referred to care. ^C^ = only 12 patients were referred for pulmonary rehabilitation because clinicians were reluctant to do this until TB treatment was completed.

**Table 6:** Performance of the referral systems for care in TB patients identified with co-morbidities, risk determinants and disability at the end of TB treatment.

Category	Variable	At end of TB Treatment	Already Receiving Regular Care	Need for Referral to Care	Referred to Care		Referred to Care		In Receipt of Care One Month after End of TB Treatment	
							Elsewhere in Another Facility	On Site at Same Facility		
		Number	Number	Number	Number	(%) ^A^	Number	Number	Number	(%) ^B^
Total cohort	Treatment success	586	52	283	231	(81.6)	57	174	143	(61.9)
Co- morbidities	Diabetes Mellitus	73	14	59	59	(100)	11	48	54	(91.5)
	Previous Diabetes	68	12	56	56	(100)	8	48	52	(92.9)
	New Diabetes	5	2	3	3	(100)	1	2	2	(66.7)
	High blood pressure	32	3	29	29	(100)	5	24	26	(89.7)
	MHD	4	0	4	4	(100)	4	0	0	(0)
Risk Determinants	Cigarette smoker	27	0	27	27	(100)	0	27	24	(88.9)
	Excess alcohol use	6	0	6	6	(100)	0	6	6	(100)
	Exposure to silica	5	0	5	5	(100)	5	0	5	(100)
	Malnutrition	95	14	81	81	(100)	0	81	69	(85.2)
Disability	6MWT < 400	137	0	137	137	(100)	29	108	23	(16.8)

Footnotes: TB = tuberculosis; MHD = mental health disorder; Malnutrition defined as body mass index < 18.0; 6MWT = 6 min walking test. ^A^ = denominator is number of patients who needed referral to care. ^B^ = denominator is number of patients who were referred to care.

## Data Availability

The data that support the findings of this study are available from the principal investigator (Yuhong Liu) upon reasonable request.
